# Pre-pregnancy weight in Swedish women and the risk of gestational diabetes and subsequent type 2 diabetes—a population-based cohort study

**DOI:** 10.1016/j.eclinm.2026.103912

**Published:** 2026-04-17

**Authors:** Jon Edqvist, Annika Rosengren, Demir Djekic, Christina E. Lundberg, Karin Andréasson, Pigi Dikaiou, Martin Adiels, Maria Åberg, Naveed Sattar, Carmen Basic, Teresia Svanvik, Martin Lindgren, Erik Thunström

**Affiliations:** a Department of Molecular and Clinical Medicine, Institute of Medicine, Sahlgrenska Academy, University of Gothenburg, Sahlgrenska University Hospital, Gothenburg SE 416 85, Sweden; b Region Västra Götaland, Department of Anesthesiology, Surgery and Intensive Care, Sahlgrenska University Hospital, Sweden; c Region Västra Götaland, Department of Medicine Geriatrics and Emergency Medicine, Sahlgrenska University Hospital, Gothenburg, Sweden; d Department of Food and Nutrition and Sport Science, Faculty of Education, University of Gothenburg, Gothenburg, Sweden; e Carlanderska Hospital, Gothenburg, Sweden; f School of Public Health and Community Medicine, Institute of Medicine, Sahlgrenska Academy, University of Gothenburg, Gothenburg, Sweden; g Region Västra Götaland, Närhälsan, Gothenburg, Sweden; h School of Cardiovascular and Metabolic Health, University of Glasgow, Glasgow, UK; i Region Västra Götaland, Department of Obstetrics and Gynaecology, Södra Älvsborgs Hospital, Borås, Sweden; j Department of Obstetrics and Gynaecology, Institute of Clinical Sciences, Sahlgrenska Academy, University of Gothenburg, Goteborg, Sweden

**Keywords:** Gestational diabetes, Type 2 diabetes, Body mass index, Epidemiology, Women’s health, Gynaecology

## Abstract

**Background:**

Obesity in young women is increasing. To which extent elevated pre-pregnancy overweight and obesity with and without gestational diabetes increase long-term risk of type 2 diabetes later in life has not been quantified.

**Methods:**

In a Swedish population-based cohort study we used data from the Swedish Medical Birth Registry in 1,153,074 primiparous women included in the registry between Jan 1, 1987 and Dec 31, 2019, with body mass index (BMI) at the first antenatal care visit as a proxy for pre-pregnancy weight to examine risk for gestational diabetes. We then compared women with gestational diabetes (n = 16,870) to age matched comparators (n = 81,862) and calculated hazards for developing type 2 diabetes identified from the National Diabetes registry over a median follow-up of 9 years.

**Findings:**

Among 1,153,074 women, 21,438 (1.9%) were diagnosed with gestational diabetes. Women with pre-pregnancy BMI > 35 kg/m^2^ had an almost 10-fold risk of gestational diabetes compared to those with low-normal weight. Among women with gestational diabetes, the hazard of type 2 diabetes began to increase already at low- or normal weight, increasing nearly exponentially with rising BMI, while the increase in risk with increasing BMI among women without gestational diabetes was much less marked. No other social/pregnancy related factors improved the prediction of type 2 diabetes.

**Interpretation:**

Gestational diabetes serves as a stress test for developing type 2 diabetes, markedly amplifying risk in even women with normal pre-pregnancy weight, and with very high absolute rates in women with pre-pregnancy obesity. Future work should ascertain to which extent women with gestational diabetes have a structured follow-up after their pregnancy, and their subsequent prognosis.

**Funding:**

The Swedish Research Council; the Swedish governmental funding of clinical research (ALF); the Swedish Heart and Lung Foundation; and Diabetes Wellness.

**Research in context**

**Evidence before this study**

We used search terms in PubMed without language or date restrictions, using combinations of Medical Subject Headings (MeSH) and free-text terms found in abstract or title related to gestational diabetes and body mass index and type 2 diabetes from database inception to February 26, 2026 yielding 864 articles from which relevant papers were chosen. Further articles were found using references from the database search. Although the literature uniformly found increased risk of type 2 diabetes among women with prior gestational diabetes, quantitative estimates varied considerably by population and follow-up time. While two large meta-analyses found an increased risk of type 2 diabetes among women with gestational diabetes, cohort studies were limited in size, had short-term follow-up or did not quantify long-term risk at different levels of pre-pregnancy BMI.

**Added value of this study**

This is a first large cohort study spanning over an extended period of time, examining how level of pre-pregnancy BMI at the index date of gestational diabetes relates to subsequent type 2 diabetes over a very long follow-up. Our findings demonstrate that a diagnosis of gestational diabetes increases the risk of future type 2 diabetes markedly, with a 20-fold hazard comparing women with normal pre-pregnancy weight with and without gestational diabetes. Compared to a reference group of women with normal pre-pregnancy BMI and without gestational diabetes, women with obesity and gestational diabetes had a notably high absolute risk, with 3–4% annual risk of type 2 diabetes over an extended follow-up, while the increase in hazard and absolute risk with rising BMI in women without gestational diabetes was much less marked. Other social or pregnancy related factors beyond BMI and gestational diabetes that we examined did not strengthen the prediction of type 2 diabetes.

**Implications of all the available evidence**

Gestational diabetes is a strong predictor for future type 2 diabetes, increasing markedly with higher pre-pregnancy body mass index, while the rise in risk with increasing BMI in women who were not diagnosed with gestational diabetes was much less marked. Accordingly, pregnancy can be said to act as a stress test for later risk of developing type 2 diabetes, and this emphasizes the need for structured clinical follow-up post-pregnancy in all women diagnosed with gestational diabetes, irrespective of their pre-pregnancy weight.

## Introduction

Following worldwide trends in overweight and obesity^1^ both rising levels of body weight among women in early pregnancy and ascending trends in the incidence of gestational diabetes have been observed in recent decades.^2^^,^^3^ Gestational diabetes is associated with insulin resistance in the second or third trimester of pregnancy and linked to an increased future risk of future type 2 diabetes.^3^ Estimates of risk of subsequent type 2 diabetes in women with gestational diabetes have varied markedly. However, two large meta-analyses have shown an approximately 7-fold up to a 10-fold increased risk of type 2 diabetes in women with prior gestational diabetes compared to women without a history of gestational diabetes.^3^^,^^4^ Despite type 2 diabetes being associated with premature death and cardiovascular disease,^5^^,^^6^ post-partum screening and follow-up of women with gestational diabetes has been suboptimal.^7^^,^^8^ Recent data show that the incidence of type 2 diabetes post gestational diabetes is nearly 25 times higher than in women without gestational diabetes,^9^ emphasizing the need for a continued long-term focus on women with gestational diabetes to prevent long-term complications. Likely, the risk of type 2 diabetes increases with higher pre-pregnancy weight but from what level and to which extent, has not been systematically studied. This study addresses the association between pre-pregnancy ***body mass index (BMI)***, gestational diabetes and the risk for future type 2 diabetes.

## Methods

### Swedish registries

Swedish antenatal care is publicly funded, free of cost to the expectant mother and includes registration of weight and height as well as a screening program for the detection of gestational diabetes,^10^ and almost all pregnant women attend.^11^ Data from antenatal and delivery care is recorded in the ***Swedish Medical Birth registry (MBR)*** allowing for long-term follow-up through nationwide Swedish registries. The Swedish National Diabetes registry with high coverage of both prevalent and new diabetes cases since 1996 allows for a detailed follow-up of up to 90% of all type 2 diabetes cases. For further information regarding MBR and weight/height measurements see Supplementary method.

### Study design and subjects

**Fig. 1:**
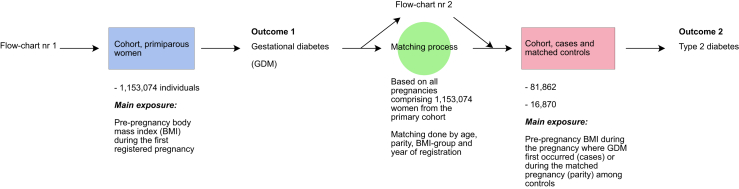
**Data analysis scheme**.

The present Swedish population-based cohort study comprised data on a cohort of primiparous women with first antenatal visits between 1987-01-01 and 2019-12-31. Data on previous pregnancies which did not pass gestational week of 22 + 0 or pregnancies that were terminated due to abortion or miscarriage were not included in the registry. We used the data to create partly overlapping study cohorts (Fig. 1), with data from an initial first cohort used for analyses regarding the overall risk of gestational diabetes by pre-pregnancy BMI, and a second cohort of cases and matched controls to assess the risk of type 2 diabetes in women with, compared to those without gestational diabetes.

### Study cohort and ethics

The initial cohort comprised 1,977,681 women registered in the MBR and after exclusions 1,153,074 primiparous women (see Supplementary Fig. S1). We excluded women with re-used personal identification numbers, those registered before 1987 (primiparous women in 1990–1991 were not included due to missing data on height and weight) and <18 years of age (n = 389,964); women with missing BMI, BMI <15 kg/m^2^ or height <140 cm or >200 cm (n = 206,996); women who had previously registered pregnancies or >4 pregnancies (n = 218,695); women who had previously recorded diabetes in the MBR (250 [ICD-9] or E10-E11 [ICD-10]) or in the national diabetes registry (any form of diabetes) (n = 8952 [see Supplementary Fig. S1])

The second cohort was based on all pregnancies among women included in the first, for the purpose of identifying a cohort with an updated cross-sectional design based on a woman’s first recorded pregnancy with gestational diabetes, and to match each pregnancy thus identified with a cohort of five control pregnancies per case matched for age, parity, exact year of registration in the MBR and BMI level (<18.5; 18.5 to <25; 25 to <30; 30 to <35; 35 and ≥35 kg/m^2^). After exclusion of women with a registered diagnosis of diabetes between baseline and the new index date post gestational diabetes (if a case had previously registered type 2 diabetes the complete matched set was removed, if a control had previously registered diabetes they were removed individually), the case cohort consisted of 16,870 women with previous gestational diabetes and the control cohort of 81,862 women without gestational diabetes. Even though controls were excluded individually, baseline data showed that matching criteria were largely met (Supplementary Fig. S2).

The study was approved by the Swedish Ethical Board Authority 2020-02226. The requirement for written informed consent was waived, as per the ethics review, owing to the register-based design that used pseudonymized data. This study is reported in accordance with the Strengthening the Reporting of Observational Studies in Epidemiology (STROBE).

### Definitions of comorbidities

We used data from the Swedish Patient Register regarding hospital in- and outpatient care with diagnoses classified according to the ***International classification of diseases (ICD)*** revision 9 and 10, together with data from the MBR which provided complementary information on medical diagnoses during pregnancy. Information regarding date and cause of death was retrieved from the Swedish Cause of Death Registry. We defined pregnancy related comorbidities and outcomes from previously validated ICD-codes.^11^ The main outcome/exposure of gestational diabetes was defined as 648 W (ICD-9) and O24.4A and O24.4B (ICD-10) in the MBR or the patient registry, while comorbidities such as preeclampsia were defined as 642E-642G (ICD-9) and O14–O15 (ICD-10), polycystic ovary syndrome (PCOS) as 256E and E28.2, gestational hypertension as 401–405, 642C (ICD-9) and O13 (ICD-10 [Supplementary Table S1]). Current smoking status was defined as current smoker and current non-smokers, while not providing information regarding current smoking status or missing data were set to unknown, where 16,710 women and 1146 women had missing values regarding current smoking status in cohort 1 and 2 respectively. In 2015 the Swedish National Board of Health and Welfare adopted the World Health Organization (WHO)-13 criteria for gestational diabetes, whereas previous diagnostics in Sweden were based on repeated random plasma glucose measurements and clinical risk factors^12^^,^^13^ and diagnostics varied by the Swedish regions^14^ and largely followed screening recommendations from the European Association for the study of Diabetes. The Swedish study Changing Diagnostic Criteria for Gestational Diabetes (CDC4G) trial observed an increase in prevalence from 2.6% up to 6.6% in a single year (2018) comparing the prior screening methods to the WHO-13 criteria, with a 3-point OGTT with fasting plasma glucose, 1-h and/or 2-h (thresholds at ≥5.1; ≥10.0; 8.5 mmol/L respectively),^12^ indicating potential under diagnostics prior to WHO-13, although the similar characteristics from the International Association of the Diabetes and Pregnancy Study Groups (IADPSG) yielded great variations in prevalence in the Hypertension Adverse Pregnancy Outcome (HAPO) study.^15^^,^^16^

### Definition of outcome

Incident type 2 diabetes after delivery was defined through the Swedish National Diabetes Registry.^17^ This registry provides roughly 90% coverage of persons in Sweden registered with type 2 diabetes.^18^ The definition of type 2 diabetes in the present study was based on the physician’s clinical assessment as well as epidemiologically defined type 2 diabetes (1. oral-/no treatment or 2. oral-/insulin treated diabetes or insulin treated diabetes with an age at onset ≥40 years or 3. the treating physician’s diagnosis of type 2 diabetes) with no latent autoimmune diabetes in adults (LADA) (Insulin treated diabetes only between 31 and 40 years at onset) or type 1 diabetes (1. Insulin treated diabetes only ≤30 years at onset or 2. the treating physician’s diagnosis of type 1 diabetes, treated with insulin only in at least 95% of the registered visits and no LADA) included. Information regarding deaths was retrieved from the Swedish Cause of Death Registry.

### Statistical analyses

The statistical analyses were divided into two periods with two baseline cohorts (landmark analysis).^19^ The first cohort consisted of primiparous women with the purpose of analysing the risk of gestational diabetes with pre-pregnancy BMI as the main exposure. Cox regression was used and models adjusted for age, year of registration (stratified as 1987–1994; 1995–1999; 2000–2004; 2005–2009; 2010–2014; 2015–2019), immigration status (Nordic or non-Nordic origin) and smoking status at baseline (stratified as current smoker, non-smoker or unknown). For confidentiality reasons data on specific country of origin, or ethnicity was not available. Sensitivity analyses were performed stratifying the risk of gestational diabetes into 1987–2014 and 2015–2019 respectively. The second analysis comprised the matched data set with pregnancies with newly diagnosed gestational diabetes and their matched control pregnancies. Cox regression was performed from the recorded gestational diabetes date until the onset of diabetes type 2 as recorded in the NDR. Individuals were censored in the case of death or in the case of a registration in NDR, without falling into the study’s definition of type 2 diabetes, or due to a registration in the patient registry (250 [ICD-9] or E10–E11 [ICD-10]), without falling into the study’s definition of type 2 diabetes. For cases occurring before the inception of the NDR in 1996 data on prevalent cases was used with the date of onset calculated from data on diabetes duration. In this analysis, gestational diabetes cases were matched to controls by BMI to investigate the excess risk of type 2 diabetes in women with gestational diabetes stratified by their BMI-category (<18.5; 18.5 to <25 [controls as the reference level]; 25 to <30; 30 to <35; ≥35 kg/m^2^). In a second analysis we used controls as a large reference group vs the above-mentioned BMI-categories. Since there was an uneven distribution of women of Nordic origin, we performed sensitivity analyses where immigration status was used as an interaction term to gestational diabetes and case/control-status. Due to non-proportionally distributed hazards when comparing gestational diabetes cases by BMI vs controls we split the follow-up time into three time periods: 0–5 years; 6–15 years; ≥16 years with ***hazard ratios (HR)*** and incidence rates presented for each time-period. We also performed crude survival analyses by BMI category stratified by gestational diabetes history and observed that within the two groups (gestational diabetes yes vs no) that the proportionality seemed acceptable within the BMI groups. Based on available data on age, year of registration (1989–1994; 1995–1999; 2000–2004; 2005–2009; 2010–2014; 2015–2019) in MBR, parity, country of birth (Nordic- or Non-Nordic origin), BMI, preeclampsia, pregnancy induced hypertension, PCOS and multiparous childbirths in connection with the current or prior pregnancies, we performed survival random forest analyses^20^ for the risk of future type 2 diabetes.

### Propensity score matching of all available pregnancies for the second updated cohort

Before matching, we excluded pregnancies with missing BMI, multiple birth pregnancies, pregnancies free from gestational diabetes that occurred before the first registered gestational diabetes among cases and pregnancies with a registered gestational diabetes diagnosis from the Swedish Patient Registry but not tied to a registered pregnancy in the MBR (see Supplementary Fig. S2 for a detailed description of the exclusion procedures). After exclusions, we used all remaining pregnancies and performed a propensity score match performed by age, exact year of registration in the MBR and BMI group, with a satisfactory matching balance displaying (see Supplementary Table S2). The propensity score match group comprised 23,800 pregnancies with gestational diabetes and 119,000 control pregnancies without gestational diabetes.

Since cases could have multiple pregnancies with or without gestational diabetes, pregnancies prior to the first gestational diabetes or pregnancies after the first registered gestational diabetes were excluded, with the complete matched set discarded (1 case and 5 controls), while the first registered pregnancy in controls was kept and concurrent pregnancies discarded on an individual basis. Thus, the information in the cases regarding age, BMI and comorbidities was updated for case with incident gestational diabetes between pregnancy 2 and 4. After exclusions controls did not appear twice. Baseline data showed that matching for the chosen covariates persisted, however, unbalanced in for instance immigration status, which was not part of the propensity score matching due to too few individuals.

### Survival random forest

The matched data set was split into training- and test data 80% and 20% respectively. The model was tuned with the inbuilt function in R-package randomForestSRC^20^ into using mtry = 4 and nodesize = 50, while we estimated a number of 500 trees with log-rank splitting as sufficient. C-index showed 0.89 for the training and test data respectively which affirmed the model as not being overfit. Variable importance was retrieved by the Breiman-Cutler method (permutation). The model displayed survival estimates for cases vs controls respectively, adjusted for the covariates included in the model. Partial dependence plots were created from the main model and showcased the estimated probability of type 2 diabetes based on newly diagnosed gestational diabetes by BMI (5 years; 15 years; and 30 years of follow-up time). All statistical analyses were performed with the software R (version 4.3.0).

### Role of the funding source

The funding sources had no role in the design and conduct of the study; collection, management, analysis, and interpretation of the data; preparation, review, or approval of the manuscript; and decision to submit the manuscript for publication.

## Results

After exclusions, the cohort for the first analysis included 1,153,074 primiparous women. Baseline characteristics stratified by pre-pregnancy BMI beginning at <18.5 kg/m^2^ and increasing by incremental steps of 2.5 up to 45 kg/m^2^ or higher are shown in Table 1. Overall, the mean age was 28.0 (SD 4.9) years, slightly lower in the two leanest categories. Women in the lower BMI range <18.5 to <22.5 kg/m^2^ comprised a higher proportion of women with non-Nordic origin compared to other BMI categories, while among those with a BMI of 35 kg/m^2^ or higher nearly 90% were of Nordic origin. Higher BMI estimates were more common in recent years. The proportion with multiple birth pregnancies was overall 1.4%, rising slightly along with increasing BMI, while smoking status varied between the BMI categories, and was most common in women with low or high BMI. Pregnancy related comorbidities were more common in women with a high BMI. Among the 1,153,551 women included at baseline, 21,438 (1.9%) were diagnosed with gestational diabetes during the index pregnancy, while 16,870 (1.5%) were eligible for follow-up of the risk for type 2 diabetes.

**Table 1:** Baseline characteristics among primiparous women by body mass index (kg/m^2^).

Variable	Overall	<18.5	18.5 to <20	20 to <22.5	22.5 to <25	25 to <27.5	27.5 to <30	30 to <35	35 to <40	40 to <45	≥45
Individuals–n	1,153,074	35,526	108,101	353,860	303,882	164,392	84,233	74,646	21,416	5600	1418
Age—mean (SD)	28.0 (4.9)	26.4 (4.8)	27.4 (4.8)	28.1 (4.8)	28.3 (4.9)	28.3 (5.0)	28.2 (5.2)	28.1 (5.2)	28.0 (5.1)	28.1 (5.0)	28.4 (5.1)
Year of registration–n (%)											
1987–1994	197,346 (17.1)	7283 (20.5)	23,234 (21.5)	72,267 (20.4)	51,991 (17.1)	23,231 (14.1)	9888 (11.7)	7742 (10.4)	1470 (6.9)	204 (3.6)	36 (2.5)
1995–1999	149,497 (13.0)	4731 (13.3)	14,426 (13.3)	47,982 (13.6)	40,626 (13.4)	21,131 (12.9)	9968 (11.8)	8005 (10.7)	2090 (9.8)	461 (8.2)	77 (5.4)
2000–2004	175,369 (15.2)	4810 (13.5)	15,168 (14.0)	53,169 (15.0)	48,008 (15.8)	26,114 (15.9)	12,816 (15.2)	10,992 (14.7)	3204 (15.0)	854 (15.2)	234 (16.5)
2005–2009	206,775 (17.9)	6004 (16.9)	18,687 (17.3)	62,182 (17.6)	54,683 (18.0)	30,120 (18.3)	15,702 (18.6)	13,725 (18.4)	4147 (19.4)	1230 (22.0)	295 (20.8)
2010–2014	224,227 (19.4)	6869 (19.3)	20,106 (18.6)	64,945 (18.4)	57,822 (19.0)	32,743 (19.9)	18,047 (21.4)	16,924 (22.7)	4995 (23.3)	1387 (24.8)	389 (27.4)
2015–2019	199,860 (17.3)	5829 (16.4)	16,480 (15.2)	53,315 (15.1)	50,752 (16.7)	31,053 (18.9)	17,812 (21.1)	17,258 (23.1)	5510 (25.7)	1464 (26.1)	387 (27.3)
Non-Nordic origin-n (%)	197,299 (17.1)	10,688 (30.1)	21,765 (20.1)	58,311 (16.5)	49,956 (16.4)	27,583 (16.8)	14,471 (17.2)	11,289 (15.1)	2538 (11.9)	556 (9.9)	142 (10.0)
Newly diagnosed conditions–n (%)											
Multiple birth	16,673 (1.4)	363 (1.0)	1295 (1.2)	4842 (1.4)	4527 (1.5)	2598 (1.6)	1405 (1.7)	1199 (1.6)	343 (1.6)	81 (1.4)	20 (1.4)
Essential hypertension	7641 (0.7)	85 (0.2)	325 (0.3)	1236 (0.3)	1531 (0.5)	1221 (0.7)	955 (1.1)	1375 (1.8)	573 (2.7)	227 (4.1)	113 (8.0)
Gestational hypertension	32,500 (2.8)	430 (1.2)	1572 (1.5)	6442 (1.8)	7398 (2.4)	5687 (3.5)	3881 (4.6)	4659 (6.2)	1685 (7.9)	542 (9.7)	204 (14.4)
Preeclampsia	58,472 (5.1)	1061 (3.0)	3405 (3.1)	12,563 (3.6)	13,910 (4.6)	9928 (6.0)	6345 (7.5)	7265 (9.7)	2857 (13.3)	854 (15.2)	284 (20.0)
Polycystic ovary syndrome	14,292 (1.2)	212 (0.6)	902 (0.8)	3120 (0.9)	3061 (1.0)	2200 (1.3)	1655 (2.0)	2101 (2.8)	824 (3.8)	185 (3.3)	32 (2.3)
Smoking at baseline	108,710 (9.6)	4432 (12.8)	10,963 (10.4)	30,810 (8.9)	26,007 (8.7)	15,346 (9.5)	8747 (10.6)	8773 (12.0)	2724 (13.0)	712 (13.0)	196 (14.1)

**Fig. 2:**
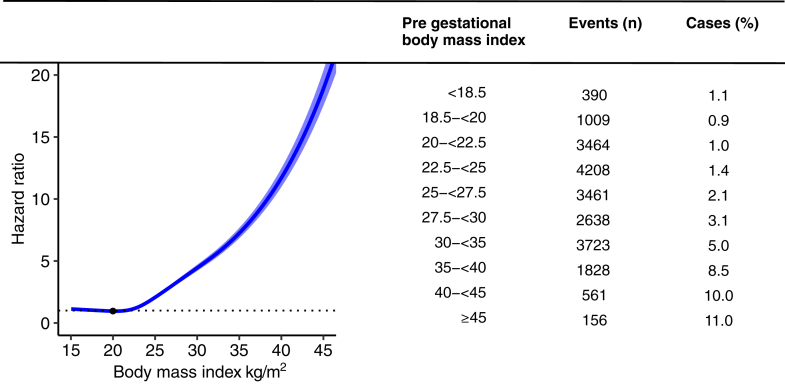
**Risk of gestational diabetes by pre-pregnancy body mass index (BMI) in primiparous women.** Models were based on Cox regression. Panel A, Hazard ratio for the risk of gestational diabetes by pre gestational BMI. Modelled as cubic spline with 4 knots adjusted for age at registration, year of registration, immigration status and current smoking status. Reference BMI 20 kg/m^2^. Light blue = Confidence interval 95%. Women were followed until an event of gestational diabetes or the last registered date of delivery.

Fig. 2 displays a strong relationship between early pregnancy BMI and the risk of future gestational diabetes (median follow-up time = 3 years) starting to increase at BMI ≥22.5 kg/m^2^ with a near linear and notable increase in incidence and HR with higher BMI levels, using BMI <20 kg/m^2^ as the reference. Women with high-normal BMI (22.5 to <25 kg/m^2^) had a slightly but significantly increased risk of gestational diabetes while women with severe obesity, or BMI >35 kg/m^2^ had an about 10-fold risk compared to the reference level. Supplementary Fig. S3 shows that the relation between early pregnancy BMI and the risk of future gestational diabetes was similar for the two periods, however, with generally higher HRs 2015–2019 than for 1987–2014. Due to limited power, it was not possible to ascertain whether there was a difference between the two periods with respect to future hazard of type 2 diabetes.

To examine the hazard of type 2 diabetes following a diagnosis of gestational diabetes, with pre-pregnancy BMI for the index pregnancy as the main exposure, we created a comparison cohort of randomly selected control cases without gestational diabetes matched for birth year, and with the baseline date set to match the date of the gestational diabetes diagnosis of the cases. The baseline characteristics for the second cohort are presented in Table 2. Women with gestational diabetes had more multiple pregnancies, were more frequently born outside the Nordic countries and more frequently had PCOS, essential and pregnancy-induced hypertension. Among the cases, 11.5% had been diagnosed with pre-eclampsia with the corresponding estimate among controls 7.8%, while the proportion of multiple births was similar between the two groups. Both essential and pregnancy-induced hypertension, as well as pre-eclampsia, rose with increasing BMI in cases and controls. Among control women without gestational diabetes and BMI≥35 kg/m^2^ 14.8% were diagnosed with preeclampsia, with the corresponding proportion among those with gestational diabetes 22.1%.

**Table 2:** Baseline characteristics among women with gestational diabetes (cases) and matched controls by body mass index (kg/m^2^).

	Overall	<18.5	18.5 to <25	25 to <30	30 to <35	≥35
Controls						
Individuals–n	81,862	1124	29,187	23,823	15,513	12,215
Age—mean (SD)	30.9 (5.3)	28.5 (5.3)	30.7 (5.1)	31.1 (5.3)	31.0 (5.5)	30.9 (5.4)
Updated year of registration–n (%)						
1987–1994	8083 (9.9)	202 (18.0)	4389 (15.0)	2169 (9.1)	928 (6.0)	395 (3.2)
1995–1999	7488 (9.1)	108 (9.6)	3194 (10.9)	2289 (9.6)	1157 (7.5)	740 (6.1)
2000–2004	10,721 (13.1)	156 (13.9)	3885 (13.3)	3310 (13.9)	1951 (12.6)	1419 (11.6)
2005–2009	15,056 (18.4)	229 (20.4)	5552 (19.0)	4298 (18.0)	2638 (17.0)	2339 (19.1)
2010–2014	19,373 (23.7)	226 (20.1)	6110 (20.9)	5777 (24.2)	4132 (26.6)	3128 (25.6)
2015–2019	21,141 (25.8)	203 (18.1)	6057 (20.8)	5980 (25.1)	4707 (30.3)	4194 (34.3)
Updated number of pregnancies–n (%)						
1	49,663 (60.7)	847 (75.4)	19,001 (65.1)	13,998 (58.8)	8863 (57.1)	6954 (56.9)
2	22,560 (27.6)	223 (19.8)	7405 (25.4)	6696 (28.1)	4601 (29.7)	3635 (29.8)
3	7961 (9.7)	48 (4.3)	2412 (8.3)	2564 (10.8)	1666 (10.7)	1271 (10.4)
4	1678 (2.0)	6 (0.5)	369 (1.3)	565 (2.4)	383 (2.5)	355 (2.9)
Nordic born–n (%)	13,519 (16.5)	325 (28.9)	4786 (16.4)	4167 (17.5)	2595 (16.7)	1646 (13.5)
Previously/newly diagnosed conditions–n (%)						
Multiple birth	1696 (2.1)	18 (1.6)	554 (1.9)	509 (2.1)	349 (2.2)	266 (2.2)
Essential hypertension	1334 (1.6)	3 (0.3)	179 (0.6)	254 (1.1)	352 (2.3)	546 (4.5)
Gestational hypertension	4202 (5.1)	16 (1.4)	716 (2.5)	1070 (4.5)	1160 (7.5)	1240 (10.2)
Preeclampsia	6359 (7.8)	29 (2.6)	1228 (4.2)	1626 (6.8)	1674 (10.8)	1802 (14.8)
Polycystic ovary syndrome	1661 (2.0)	8 (0.7)	309 (1.1)	417 (1.8)	449 (2.9)	478 (3.9)
Smoking status—n (%)						
Non-smoker	73,475 (89.8)	980 (87.2)	26,604 (91.2)	21,491 (90.2)	13,720 (88.4)	10,680 (87.4)
Smoker	6661 (8.1)	129 (11.5)	2009 (6.9)	1842 (7.7)	1421 (9.2)	1260 (10.3)
Unknown	1726 (2.1)	15 (1.3)	574 (2.0)	490 (2.1)	372 (2.4)	275 (2.3)
Cases						
Individuals–n	16,870	240	5940	4884	3274	2532
Age—mean (SD)	31.0 (5.3)	28.9 (5.5)	30.7 (5.2)	31.2 (5.3)	31.3 (5.6)	30.9 (5.3)
Updated year of registration–n (%)						
1987–1994	1658 (9.8)	42 (17.5)	881 (14.8)	439 (9.0)	197 (6.0)	99 (3.9)
1995–1999	1534 (9.1)	23 (9.6)	646 (10.9)	468 (9.6)	243 (7.4)	154 (6.1)
2000–2004	2214 (13.1)	33 (13.8)	787 (13.2)	678 (13.9)	406 (12.4)	310 (12.2)
2005–2009	3044 (18.0)	49 (20.4)	1123 (18.9)	878 (18.0)	551 (16.8)	443 (17.5)
2010–2014	3951 (23.4)	48 (20.0)	1240 (20.9)	1180 (24.2)	867 (26.5)	616 (24.3)
2015–2019	4469 (26.5)	45 (18.8)	1263 (21.3)	1241 (25.4)	1010 (30.8)	910 (35.9)
Updated number of pregnancies–n (%)						
1	9897 (58.7)	174 (72.5)	3815 (64.2)	2801 (57.4)	1770 (54.1)	1337 (52.8)
2	4788 (28.4)	47 (19.6)	1540 (25.9)	1408 (28.8)	994 (30.4)	799 (31.6)
3	1771 (10.5)	16 (6.7)	509 (8.6)	546 (11.2)	396 (12.1)	304 (12.0)
4	414 (2.5)	3 (1.2)	76 (1.3)	129 (2.6)	114 (3.5)	92 (3.6)
Nordic born–n (%)	5612 (33.3)	101 (42.1)	2106 (35.5)	1846 (37.8)	1028 (31.4)	531 (21.0)
Previously/newly diagnosed conditions–n (%)						
Multiple birth	365 (2.2)	6 (2.5)	126 (2.1)	102 (2.1)	74 (2.3)	57 (2.3)
Essential hypertension	423 (2.5)	1 (0.4)	62 (1.0)	89 (1.8)	115 (3.5)	156 (6.2)
Gestational hypertension	1155 (6.8)	5 (2.1)	194 (3.3)	302 (6.2)	314 (9.6)	340 (13.4)
Preeclampsia	1947 (11.5)	4 (1.7)	365 (6.1)	524 (10.7)	495 (15.1)	559 (22.1)
Polycystic ovary syndrome	557 (3.3)	1 (0.4)	98 (1.6)	153 (3.1)	160 (4.9)	145 (5.7)
Smoking status–n (%)						
Non-smoker	15,037 (89.1)	216 (90.0)	5357 (90.2)	4397 (90.0)	2893 (88.4)	2174 (85.9)
Smoker	1480 (8.8)	21 (8.8)	453 (7.6)	382 (7.8)	321 (9.8)	303 (12.0)
Unknown	353 (2.1)	3 (1.2)	130 (2.2)	105 (2.1)	60 (1.8)	55 (2.2)

**Fig. 3:**
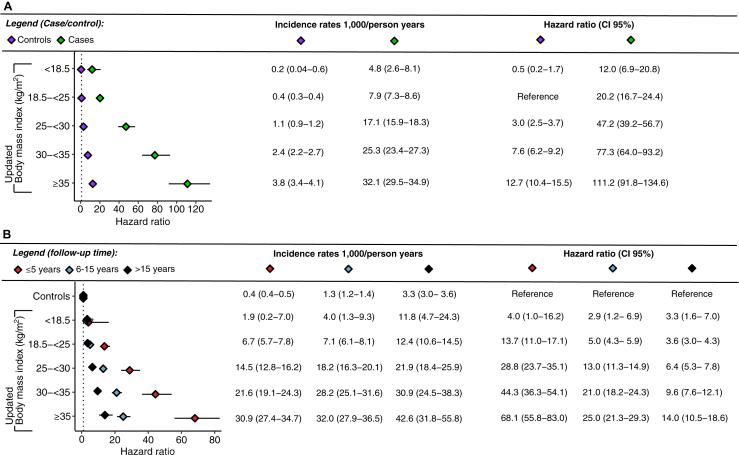
**Risk of subsequent type 2 diabetes by body mass index (BMI) matched by BMI vs BMI and BMI vs controls without gestational diabetes.** Models were based on Cox regression. Panel A, Hazard ratio for the risk of type 2 diabetes post gestational diabetes by BMI. BMI match by five BMI categories with women without any history of gestational diabetes. BMI 18.5 to <25 kg/m^2^ among controls were used as the reference group for each BMI category. Panel B, Hazard ratio for the risk of type 2 diabetes post gestational diabetes by pre gestational BMI compared to matched controls (reference group). Time was split into 3 periods: ≤5 years; 6–15 years; >15 years with corresponding incidence rates and hazard ratio presented for each period respectively. In panels A and B BMI, age and year of registration were updated if the parity was 2–4 (Primiparous women with GDM or primiparous controls, kept their baseline data). Panels A and B were adjusted for age at registration, year of registration, immigration status and current smoking status. CI = Confidence interval.

Fig. 3A shows the risk of incident type 2 diabetes by pre-pregnancy BMI and presence of gestational diabetes for the index pregnancy over the complete follow-up period (median follow-up time = 9 years). With women with low-normal weight and without gestational diabetes as reference, hazards of future diabetes rose from approximately 12-fold in women with BMI ≥ 35 kg/m^2^ without gestational diabetes, to about 100 times greater in the same BMI category with gestational diabetes. With respect to absolute risk, women without gestational diabetes had comparatively low incidence of type 2 diabetes, at ∼2 and ∼4 per 1000 person-years even among the two highest BMI categories, which should be compared with 25.3 and 32.1 per 1000 person-years for the same BMI categories in women with gestational diabetes, or an approximate incidence rate of 3% per year (Fig. 3A).

When the entire control group was used as a reference (Fig. 3B), we observed a gradual increase in the hazard of type 2 diabetes with increasing BMI, which was highest in the short-term perspective (0–5 years) with an almost 70-fold increase compared to the total group of women without gestational diabetes but the risk was still increased by a factor of about 25 after 5 years and 14-fold after 11 years. Although the relative hazard of type 2 diabetes in women without gestational diabetes decreased with time, the incidence rates were constantly high for women with obesity and gestational diabetes, even after over 15 years of follow-up.

Supplementary Figs. S4 and S5 survival curves show that women with BMI ≥35 kg/m^2^ and gestational diabetes had an estimated probability of at least 50% of developing type 2 diabetes after 30 years regardless of immigration status. Among women without previous exposure of gestational diabetes, risks of type 2 diabetes were largely similar regardless of country of birth.

**Fig. 4:**
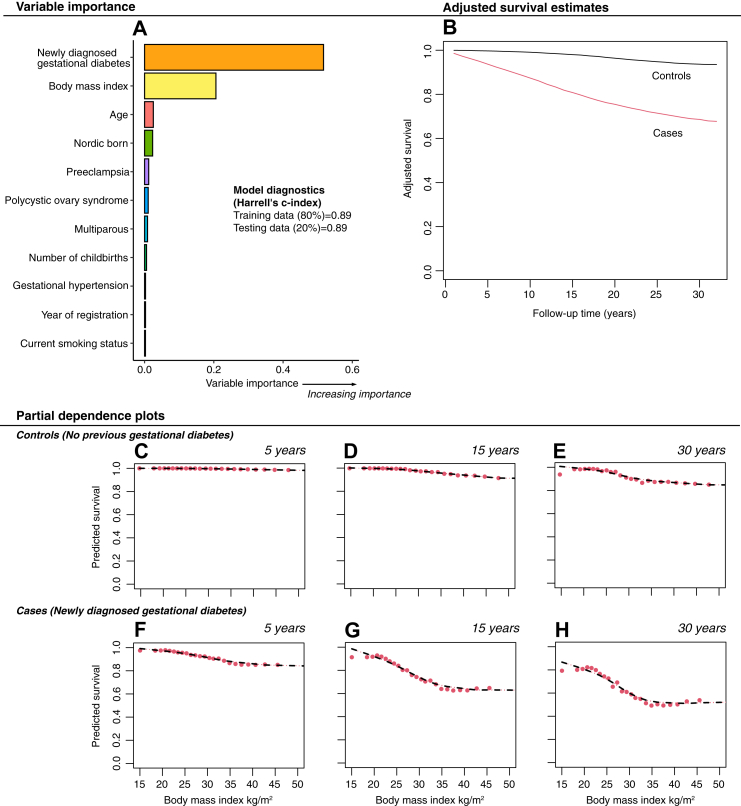
**Prediction model based on survival random forests among women with gestational diabetes and matched controls for the risk of incident type 2 diabetes.** Models were based on survival Random forests. Panel A, Survival random forest model based on training data (80%), evaluated on test data (20%). Panel B, Survival estimates from the survival Random forest main model, adjusted for the covariates in panel A. Panels C–H, partial dependence plots displaying estimated survival based on body mass index at 5 years of follow-up; 15 years of follow-up and 30 years of follow-up. Models are based on the survival Random forest main model in Panel A, adjusted for the covariates in panel A.

Fig. 4 displays results originating from the tuned survival random forest model. Even though we added other pregnancy related comorbidities, not prevalent in the main models, factors such as age, immigration status, multiple childbirth, number of pregnancies, preeclampsia and pregnancy-induced hypertension were largely insignificant compared to previous gestational diabetes and BMI with respect to predicting future type 2 diabetes. With a relatively strong prediction (c-index = 0.89 [0.5–1]), we identified that gestational diabetes, and BMI both had a substantial impact on the prediction of incident type 2 diabetes. Even though we identified a higher risk of type 2 diabetes in immigrants (Supplementary Figs. S4 and S5), immigration status (non-Nordic origin) did not have any meaningful independent impact on the prediction accuracy. Adjusted partial dependence plots reaffirmed the substantial impact of obesity and previous gestational diabetes for the prediction of incident type 2 diabetes, with no other factors playing any substantial role.

## Discussion

In this observational study on over 1.1 million primiparous women, about 1.9% were diagnosed with gestational diabetes and 1.5% followed for the risk of type 2 diabetes. The risk increased monotonically with BMI, from about 1% in women with normal weight to 5% in women with BMI 30 to 35, and ∼10% in women with BMI 40 kg/m^2^ or higher. The subsequent risk of type 2 diabetes associated with gestational diabetes in women with obesity was estimated at about 2–3% annually and an estimated probability of type 2 diabetes of at least 50% after 30 years. After taking other factors into consideration, gestational diabetes was the strongest predictor of type 2 diabetes, followed by pre-pregnancy BMI, essentially serving as a stress test for future diabetes in young women.

Systematic information on how gestational diabetes was diagnosed over the extended time frame of the study, in the many prenatal care units involved was unavailable to us. Likely, many units, particularly early in the study, will have settled for detection of glucosuria using dipsticks, while blood samples and oral glucose tolerance tests (OGTTs) became increasingly more common over the period. Likely, gestational diabetes was underdiagnosed during the first part of the study but to which extent is unknown. Still, those detected through the less sensitive methods of the early part of the study may have contributed to overestimation of risk in this group, while the low rates among those not detected will have been less influenced.

Since average BMI in young Swedish women has increased substantially since 1980^2^ this could potentially argue for a routine gestational diabetes screening using OGTT since the risk of gestational diabetes was seen to increase even in women with relatively low BMI. Universal screening might also resolve the potential problem of stigmatizing women with obesity which has been cited as a problem for the midwife to propose OGTT in women with risk factors for gestational diabetes, including obesity. Still, the potential benefits of gestational diabetes screening remain debated,^21^^,^^22^ even in the US where the prevalence of gestational diabetes during pregnancy may be as high as 9.4%,^22^ Lifestyle changes in high-risk pregnant women may reduce risk of gestational diabetes substantially according to a small Finnish study^23^ but to which extent this intervention could delay or prevent the development of type 2 diabetes in the long term remains unknown.

The combination of obesity and gestational diabetes confers markedly elevated risk of type 2 diabetes in the short-term, as well as in the long-term perspective, and even though the relative risk decreased over time due to an increased risk in women with no previous gestational diabetes, up to at least 50% of women with a history of gestational diabetes and BMI ≥35 kg/m^2^ were later diagnosed with type 2 diabetes. The findings of the present study highlight the importance of clinical awareness regarding women with a history of gestational diabetes by focusing on traditional type 2 diabetes prevention such as lifestyle interventions^24^ and an early strict risk factor control among women with manifest type 2 diabetes to prevent late complications. Women with type 2 diabetes and poor risk factor control have a multiple-fold increased risk of incident cardiovascular disease or death compared to the general population.^5^^,^^6^^,^^25^ Treatment post-partum differs significantly by country, where a comparison between the American Diabetes Association, the American College of Obstetricians and Gynaecologists, and the National Institute for Health and Clinical Excellence (NICE) display widely varying recommendations.^26^ However, all guidelines recommend lifestyle interventions,^26^ which, given the high long-term incidence more than a decade post gestational diabetes found in this study, should be implemented on all women with gestational diabetes regardless of weight and risk factor profile. Optimal time and method of long-term follow-up to avoid type 2 diabetes should be further explored in future research. Even so, to which extent women with past gestational diabetes are being followed up in Sweden to detect type 2 diabetes is not documented, because of a lack of data from primary care.

Women with gestational diabetes, even those with normal pre-pregnancy weight, may have chronic metabolic- and beta-cell deficiencies, leading to hyperglycemia and subsequent risk of type 2 diabetes.^27^ Thus, our results indicate a continued focus on prevention and long-term follow-up for women with gestational diabetes to prevent and detect future incident type 2 diabetes. The most obvious and proven effective intervention is aggressive lifestyle intervention,^28^ and dietary change.^29^ Insulin treatment may be introduced^30^ or oral components as administration of metformin^31^ or troglitazone.^32^^,^^33^ Effects of the novel GLP-1 receptor agonists, developed as an anti-diabetic drug, which also can be used for weight reduction have not yet been clarified. Our prediction model which performed well on unseen test data with relatively few and easy measured variables, implies the need of a structured follow-up of all women with gestational diabetes, with at least yearly follow-up of blood glucose and HbA1c.

Strengths of the study were the large cohort of women consisting of 1,153,074 women with a recorded pre pregnancy BMI during the first registered pregnancy from which we identified 16,870 pregnancies with incident gestational diabetes and 81,862 matched control pregnancies. Because of the availability and nationwide coverage of registries, most cases of gestational diabetes are likely to have been captured, as well as type 2 diabetes during follow-up.

There are also some limitations to this study. Primarily, while most women underwent multiple tests for potential gestational diabetes, the assessment and detection process lacked uniformity. The use of OGTTs, although recommended for women perceived to be at high risk was introduced late^11^ and the Swedish local compliance rates to guidelines have been low.^34^ The rate of gestational diabetes is low compared to other countries, which could be due to insufficient testing, with only the most severe cases being detected. Still, Sweden, similar to other northern European countries, has low diabetes rates in women (about 4%) ranking among the lowest in the world and which do not seem to be increasing.^35^ The following of WHO-13 guidelines for detecting gestational diabetes has been observed to substantially increase the Swedish prevalence of gestational diabetes,^12^ which would imply a possible slight overestimation of the risk for type 2 diabetes in the present study, if gestational diabetes in Sweden has been underdiagnosed. However, our present HR was high with increased absolute risks of type 2 diabetes at a short-, middle- and long-term time span which would probably imply careful follow-up in women with impaired glucose control during pregnancy. There is also a need for future research with a more clearly defined population and selection of an appropriate control population with a similar follow-up regimen as women diagnosed with gestational diabetes in order to get as reliable risk ratios as possible. We also emphasize the lack of family history data in our study, a factor which might interact with BMI, gestational diabetes and type 2 diabetes, and which could be a subject for future study. Another potential limitation is the coverage of type 2 diabetes in the NDR which is approximately 90%. However, it has been previously shown that information regarding type 2 diabetes from Swedish Patient Registry is similar to that using only NDR when investigating type 2 diabetes.^17^ There may also be difficulties in retrieving accurate year for age at onset using only the Swedish Patient Registry.

Our study confirmed a high risk of developing type 2 diabetes among women diagnosed with gestational diabetes. Concomitant overweight, obesity and severe obesity further increased the hazard. However, women with pre-pregnancy weight within the normal range also had an excess risk of type 2 diabetes compared to normal weight individuals without prior gestational diabetes. These results suggest that both maternal BMI and the occurrence of gestational diabetes need to be considered in determining risk for, and therefore efforts targeting prevention of, future type 2 diabetes.

## Contributors

JE initiated the study, designed the analysis plan, performed statistical analyses, and led manuscript writing. ET initiated the data collection and obtained ethical permission. JE and MA performed statistical analyses, accessed and verified the underlying data. ET, AR, and MA contributed to study conceptualization, analytical planning, and initial manuscript drafting. JE, MA and ET had full access to all the data in the study and take responsibility for the integrity of the data and the accuracy of the data analysis. JE, AR, DD, CEL, KA, PD, MA, MÅ, NS, CB, TS, ML, ET all contributed in terms of critical revision of the manuscript for important intellectual content. All authors read and approved the final version of the manuscript. AR, ET, JE obtained the funding for the manuscript.

## Data sharing statement

The data used for this study are not available from the author, for legal reasons. However, they are available from the Swedish National Board of Health and Welfare, Statistics Sweden, and Region Västra Götaland for researchers, after fulfilling legal and regulatory requirements, including, but not limited to, permission from the Swedish Ethical Review Authority.

## Declaration of interests

N.S. declares consulting fees or speaker honoraria, or both, from Abbott Laboratories, Afimmune, Amgen, AstraZeneca, Boehringer Ingelheim, Eli Lilly, Hanmi Pharmaceutical, Janssen, Merck Sharp & Dohme, Novartis, Novo Nordisk, Pfizer, and Sanofi and grant support paid to his university from AstraZeneca, Boehringer Ingelheim, Novartis, and Roche Diagnostics. No other potential conflicts of interest relevant to this article were reported. C.B. declares consulting fees or speaker honoraria, or both, from AstraZeneca, Boehringer Ingelheim, Novartis, BMS, Vifor Pharma, Bayer. C.B. declares participation on Advisory Bord for AstraZeneca, Boehringer Ingelheim and Bayer.
