# Supplementary material for: Pre-pregnancy weight in Swedish women and the risk of gestational diabetes and subsequent type 2 diabetes—a population-based cohort study
Source: eClinicalMedicine. 2026 Apr 17;95:103912. doi: 10.1016/j.eclinm.2026.103912 (PMC13099346; doi:10.1016/j.eclinm.2026.103912)
Supplement: Supplementary methods, Figs. S1–S5, and Tables S1 and S2 [file mmc1.docx]

**Pre-pregnancy weight in Swedish women and the risk of gestational diabetes and subsequent type 2 diabetes – a population-based cohort study**

Supplementary material

**Supplementary method**

**Medical birth registry**

Antenatal care in Sweden is publicly funded and free of charge, with a very high attendance rate (99% of women bearing children) and data from all visits for pregnancies that resulted in a live birth has been recorded in the medical birth registry from 1973 and onwards. We used data from this registry to obtain information on age, height, weight, and medical conditions from the first registered antenatal visit (regardless of number of prior pregnancies), supplemented by comorbidities and deaths registered during pregnancy, in Sweden from 1987 to 2019 (last death date i.e, end of study was set to 31 December, 2020). Weight data in early pregnancy was not recorded in 1990 and 1991 and women attending antenatal care during this period were excluded from the present study. Height (self-reported) has been recorded at the first antenatal visit from 1992, while height prior to 1992 was obtained from records on weight and height at delivery. Measured weight at the first antenatal visit usually taking place early in the first trimester, has been registered since 1992 and was used as a proxy for pre-pregnancy weight in the present study, as in several previous studies.^1,2^ Until 1989, early pregnancy weight was calculated from weight at delivery and weight gain. Weight at delivery was recorded using two digits only (e.g. weight ≥100 kg was recorded as 99 kg). During the study period valid information on height and weight was recorded in about 80% and 70% of cases, respectively. There was an increase in the prevalence of overweight and obesity throughout the period. A visual inspection of trends in body weight deciles showed a larger than expected increase in body weight between 1989 and 1992. Because of this increase, considered to be caused by changes in the manner that data on weight was obtained, the weights before and including 1989 were adjusted by estimating annual weight increase within deciles from 1992–2003, which generated a nearly linear result^3^. From 1992 and onwards weight was measured during the first antenatal visit within 12 weeks of gestation (in 90% of cases) while height was self-reported^4^.

**References:**

1. Lundberg CE, Ryd M, Adiels M, Rosengren A, Björck L. Social inequalities and trends in pre-pregnancy body mass index in Swedish women. *Sci Rep* 2021; **11**(1): 12056.

2. Robertson J, Lindgren M, Schaufelberger M, et al. Body Mass Index in Young Women and Risk of Cardiomyopathy: A Long-Term Follow-Up Study in Sweden. *Circulation* 2020; **141**(7): 520-9.

3. Persson CE, Adiels M, Björck L, Rosengren A. Young women, body size and risk of atrial fibrillation. *European journal of preventive cardiology* 2018; **25**(2): 173-80.

4. The Centre for Epidemiology (EpC) at the National Board of Health and Welfare. The Swedish Medical Birth Register: A summary of content and quality. 2003. <https://www.socialstyrelsen.se/globalassets/sharepoint-dokument/artikelkatalog/ovrigt/2003-112-3_20031123.pdf>

| Table S1-ICD-codes | | |
| --- | --- | --- |
| Diagnosis | ICD-9 | ICD-10 |
| Gestational diabetes | 648W | O24.4A, O24.4B |
| Polycystic ovary syndrome | 256E | E28.2 |
| Pregnancy induced hypertension | 642D, 642X | O13 |
| Preeclampsia | 642E-642G | O14-O15 |

| **Table S2-Summary of table for matched data** | | | |
| --- | --- | --- | --- |
|  | Means Treated | Means Control | Std. Diff mean |
| Distance | 0.0296 | 0.095 | 0.0020 |
| Age | 31.3884 | 31.3841 | 0.0008 |
| Year | 2009.4021 | 2009.3586 | 0.0053 |
| Parity | 1.73 | 1.7320 | 0.0069 |
| <18.5 | 0.3205 | 0.3206 | -0.0004 |
| 18.5-<25 | 0.0122 | 0.0117 | 0.0044 |
| 25-<30 | 0.2900 | 0.2901 | -0.0001 |
| 30-<35 | 0.2048 | 0.2040 | 0.0022 |
| ≥35 | 0.1724 | 0.1736 | -0.0033 |


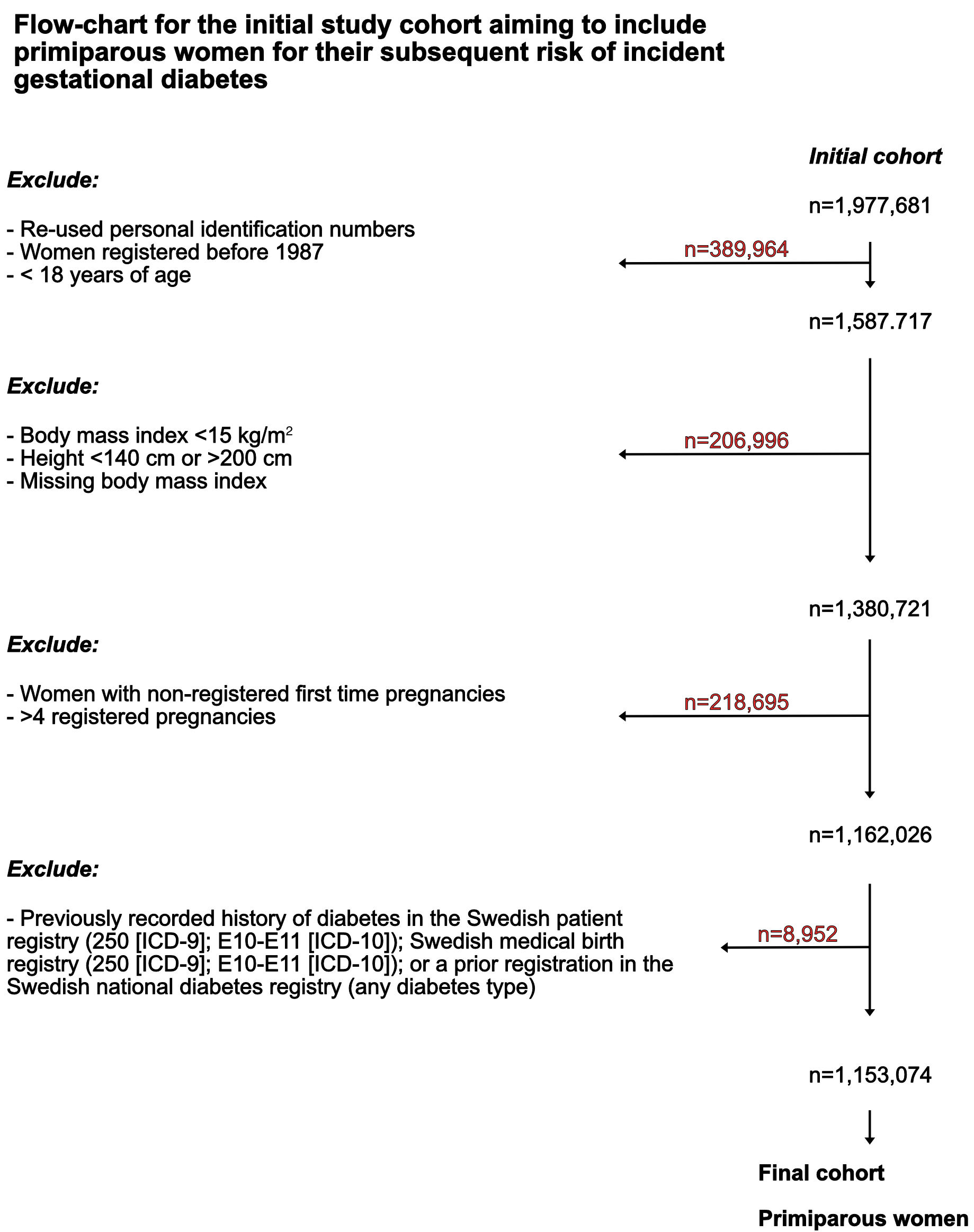


**Figure S1 - Flow chart for the main cohort based on first time pregnancies**


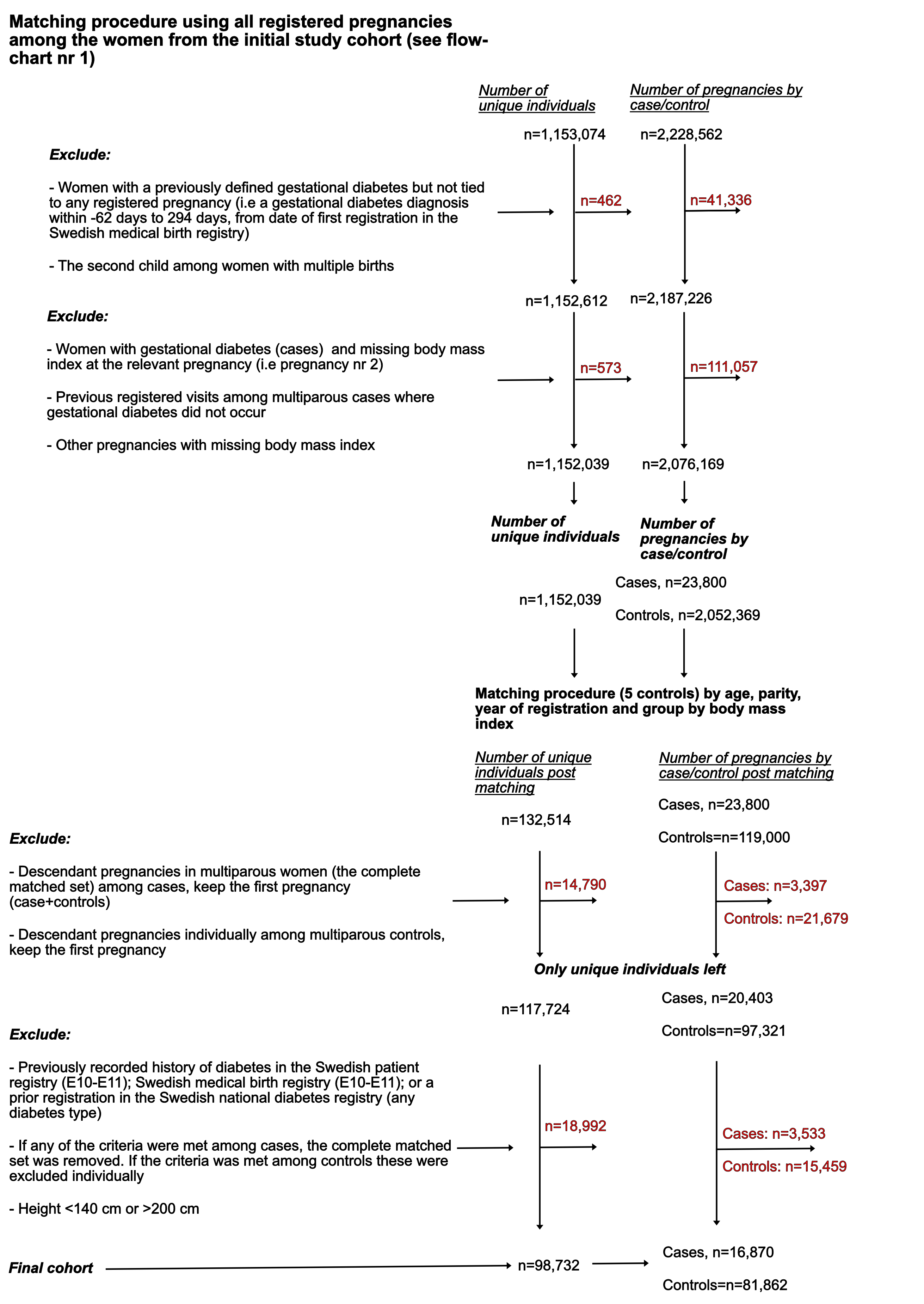


**Figure S2 - Flow chart for the matched cohort with start date based on the gestational diabetes (cases) and 5 matched controls**

The cohort initially comprised women from the initial cohort and all their pregnancies. Post the matching procedure, the cohort comprised cases with their first available GDM-pregnancy and controls matched for age, parity, year of registration and group by body mass index.


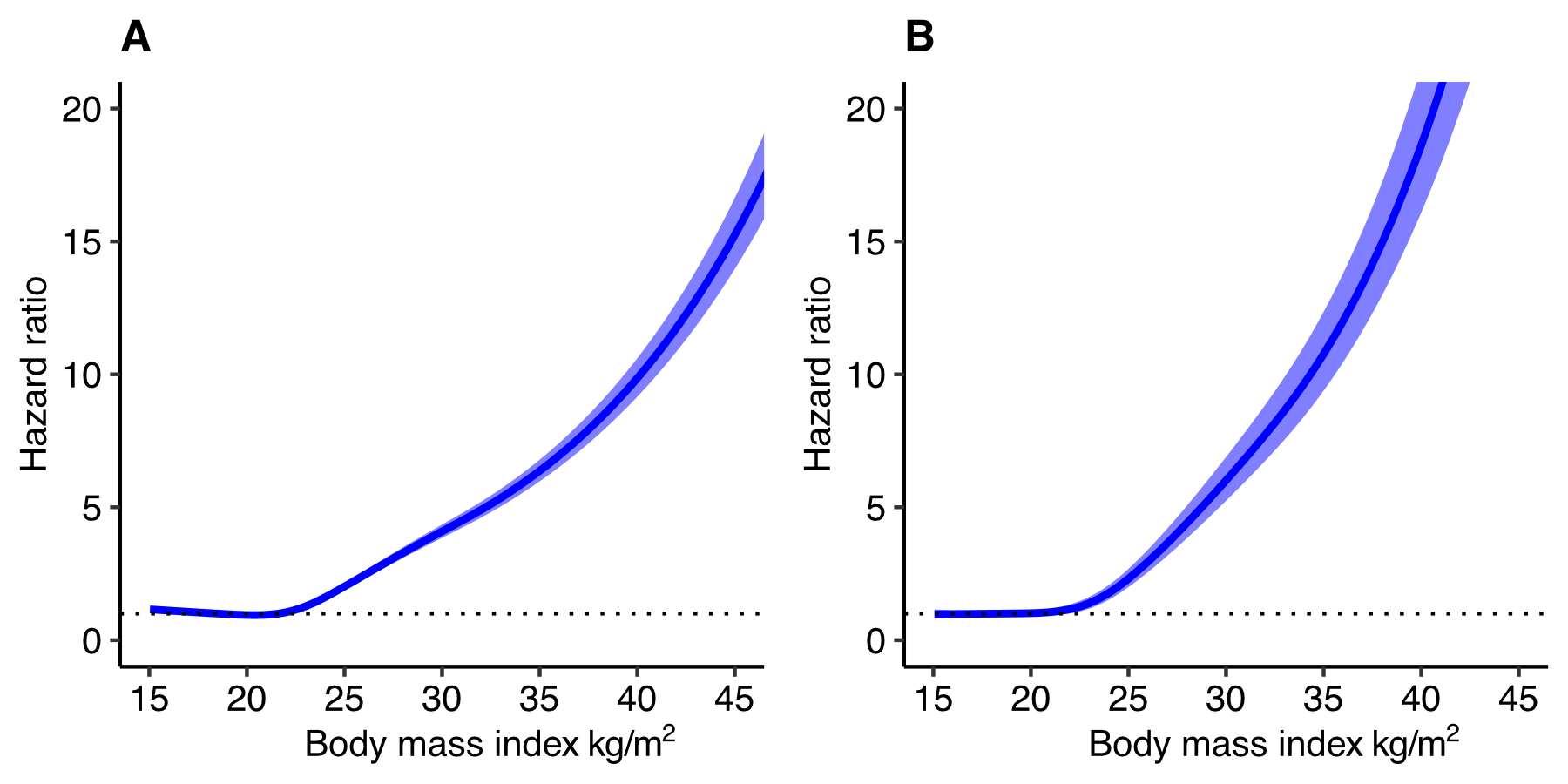


**Figure S3 - Risk of gestational diabetes by pre-pregnancy body mass index (BMI) in primiparous women stratified by time period**

Models were based on Cox regression. Panel A, Primiparous women registered between 1987-2015. Hazard ratio for the risk of gestational diabetes by pre gestational BMI. Modelled as cubic spline with 4 knots adjusted for age at registration, year of registration (1987-1994; 1995-1999; 2000-2004; 2005-2009; 2010-2014), immigration status and current smoking status. Panel B, Primiparous women registered 2015-2019. Hazard ratio for the risk of gestational diabetes by pre gestational BMI. Modelled as cubic spline with 4 knots adjusted for age at registration, immigration status and current smoking status. Reference BMI 20 kg/m^2^. Light blue=Confidence interval 95%. Women were followed until an event of gestational diabetes or the last registered date of delivery.


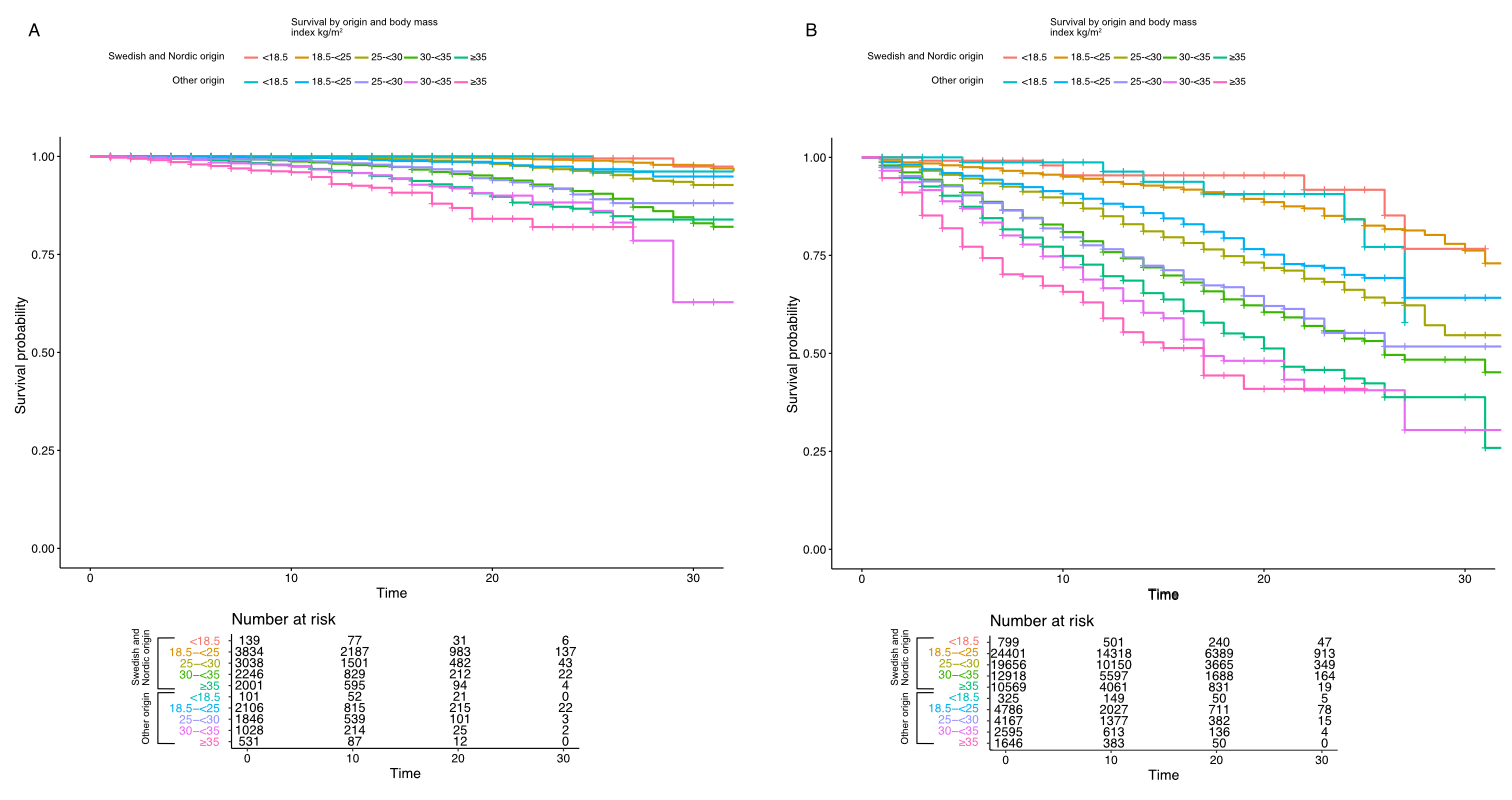


**Figure S4 - Crude survival curve for the risk of type 2 diabetes post gestational diabetes**

Panel A, Matched controls without previous gestational diabetes. Panel B, Cases with previous gestational diabetes.


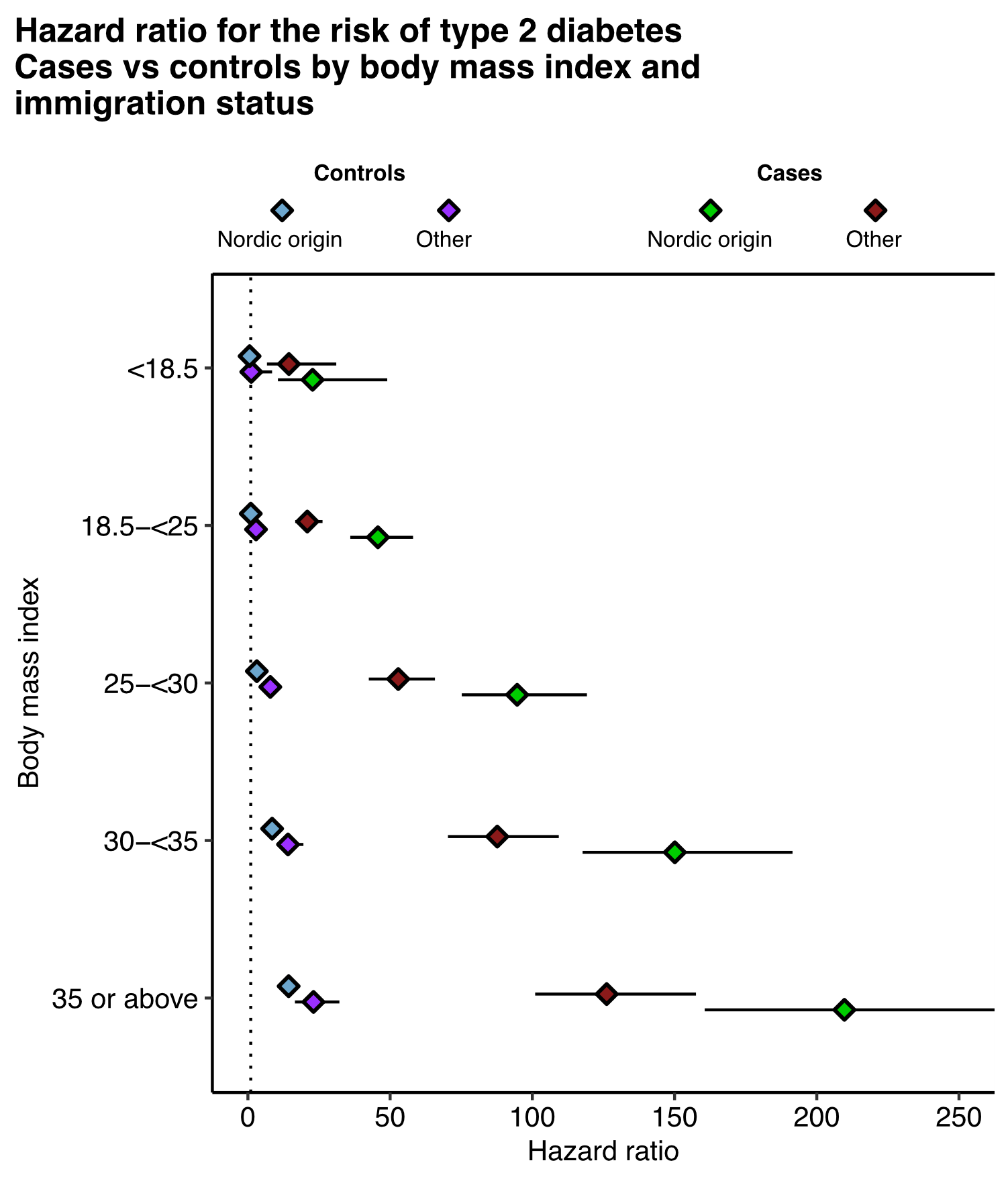


**Figure S5 - Risk of subsequent type 2 diabetes by body mass index (BMI) matched by BMI vs controls without gestational diabetes**

Hazard ratio for the risk of type 2 diabetes post gestational diabetes by BMI. BMI match by five BMI categories with women without any history of gestational diabetes. Controls with BMI 18.5-<25 kg/m^2^ and women of Nordic origin were used as the reference group for each BMI category. BMI, age and year of registration were updated if the parity was 2-4 (Primiparous women with GDM or primiparous controls, kept their baseline data). The analysis was adjusted for age at registration, year of registration and current smoking status.
